# Superior Intracellular Antioxidant Activity of an Astaxanthin-Containing Corynebacterial Extract

**DOI:** 10.3390/ijms27083638

**Published:** 2026-04-19

**Authors:** Jan Seeger, Nadja A. Henke

**Affiliations:** 1 Genetics of Prokaryotes, CeBiTec & Faculty of Biology, Bielefeld University, 33615 Bielefeld, Germany; 2 Institute of Process Engineering in Life Sciences, Karlsruhe Institute of Technology, 76131 Karlsruhe, Germany

**Keywords:** intracellular antioxidant activity, astaxanthin, keratinocytes, bioavailability

## Abstract

Astaxanthin can be derived from various sources, including petrochemical synthesis, natural sourcing from green algae, or microbial fermentation. As one of the strongest antioxidants known by nature, astaxanthin is attracting attention as an active ingredient in cosmetic products designed to protect the skin against oxidative stress. In contrast to widely performed chemical antioxidant activity assays, this study compares synthetic, algal, and corynebacterial astaxanthin in a physiologically relevant test setting: the intracellular antioxidant activity in cultured human skin cells (keratinocytes). The astaxanthin-rich corynebacterial oleoresin demonstrated superior antioxidant properties in the assay with an EC_50_ of 2.7 µM, whereas the synthetic and algal-based variants showed no significant effect. Given the potential application of such raw materials, it is therefore tempting to speculate that astaxanthin-containing corynebacterial oleoresins could serve as a natural, superior active ingredient for skin health applications in the future.

## 1. Introduction

Oxidative stress is closely linked to inflammatory processes and is therefore associated with a range of health issues ranging from cardiovascular and neurodegenerative diseases, diabetes, and cancer to skin diseases and aging [1,2,3,4]. Environmental triggers such as pollution and UV light create ubiquitous oxidative stress in biological systems [5,6,7,8]. Oxidative stress can harm cells in multiple ways, impacting different key processes associated with various locations within the cell. At the plasma membrane/cell envelope, lipid peroxidation causes membrane alterations and interference with receptor signaling [9]. Furthermore, intracellular oxidative stress can interfere with various cellular processes, including DNA repair [10] and protein folding [11,12], as well as affecting subcellular structures [13]. Antioxidants are therefore a dominant group of active ingredients [14] for various applications, ranging from nutrition to cosmetics and pharmaceuticals [15,16,17].

Astaxanthin, known as “the queen of carotenoids”, is one of the most powerful antioxidants found in nature [18]. Its excellent activity can be explained by the conjugated double bond system and the further oxyfunctionalized groups at the β-ionone rings of its structure [18,19]. The astaxanthin market share is dominated by natural astaxanthin, with a share of >600 Mio. USD in 2024 and a CAGR of 8.8% in the forecasted period till 2034 [20]. Natural variants on the markets are, for example, sources from the red yeast *Phaffia rhodozyma* [21,22,23], the bacterium *Paracoccus carotinifaciens* [24], and the green microalgae *Haematococcus pluvialis* [25]. In addition to these well-established production hosts, strain and bioprocess engineering allow us to tackle the astaxanthin market with other microbial cell factories such as *Escherichia coli* [26,27] and *Yarrowia lipolytica* [28,29]. In recent years, *Corynebacterium glutamicum* has been presented as an alternative microbial cell factory for astaxanthin production, with fermentative protocols [30,31,32] and downstream processes [33,34] having been established and optimized.

Besides their different sources, the synthesized astaxanthins differ in their stereochemistry and esterification. Synthetic astaxanthin is present as a racemate (a 1:2:1 mixture of (3S,3′S), (3S,3′R), and (3R,3′R)) in its free form [35]. Natural biosynthesis typically results in either the 3S,3′S enantiomer (dominantly algal, bacterial, and plant sources) [36,37] or the 3R,3′R enantiomer (dominantly yeast sources) [38]. Depending on the specific production host, astaxanthin may be present in its free form or esterified with fatty acids [39,40]. This means that a series of structurally different astaxanthin variants exists, which may have different functional properties. In vitro assays, such as the 2,2-diphenyl-1-picrylhydrazyl (DPPH) assay, allow the antioxidant activity to be easily determined based on the color change during quenching of the free radical DPPH in the presence of a ROS scavenger [41]. This assay is widely applied due to its easy, fast, and cheap handling. However, the interpretation of such chemical assays for real-world application is limited. Active (pharmaceutical) ingredients have to be bioavailable [42,43,44] in order to fulfill their function at the desired cellular location.

As a promising active ingredient in skincare products, topical application of astaxanthin has been shown to reduce wrinkles, improve elasticity and pigmentation [45], support wound healing [46], and mitigate UV-induced skin damage [47]. Therefore, the scope of this work is on the investigation of the antioxidant activity of three different astaxanthin sources, namely, synthetic, algal-based, and corynebacterial astaxanthin (as present in a corynebacterial natural extract), using a more physiologically relevant test setting involving human keratinocytes.

## 2. Results

### 2.1. Intracellular Antioxidant Activity Testing of Different Astaxanthins

**Figure 1:**
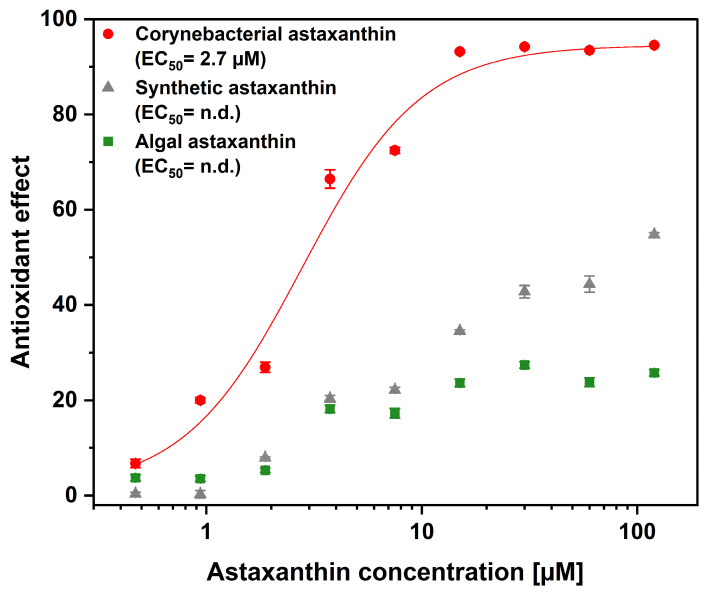
Intracellular antioxidant activity of astaxanthin from different sources. Corynebacterial astaxanthin-containing oleoresin (CA), synthetic and algal (SA) astaxanthin-containing oleoresin (AA) are shown in red, gray, and green, respectively. Values are given as mean ± standard deviation (n = 3).

The human keratinocyte cell line HaCaT is a widely used model of the epidermis and is therefore employed to investigate skin physiology and evaluate novel cosmetic ingredients [48,49]. As astaxanthin is a promising ingredient for skin applications [50], the assay was performed on human keratinocytes (HaCaT). The assay uses the fluorescent dye thiazole orange, which is taken up by the cells. When illuminated, it triggers the generation of ROS, such as singlet oxygen and hydroxyl radicals. These ROS can be neutralized by the addition of an antioxidant [51]. The results of the AOP1 assay are shown in Figure 1. The half-maximal effective concentration (EC_50_) of corynebacterial astaxanthin (CA) was determined to be 2.7 µM. By contrast, no calculable EC_50_ was obtained for algal-based astaxanthin (AA) or synthetic astaxanthin (SA), as only partial activity was observed even at the highest concentrations examined (120 µM).

### 2.2. De-Esterification of Algal-Based Astaxanthin and Its Intracellular Activity

**Figure 2:**
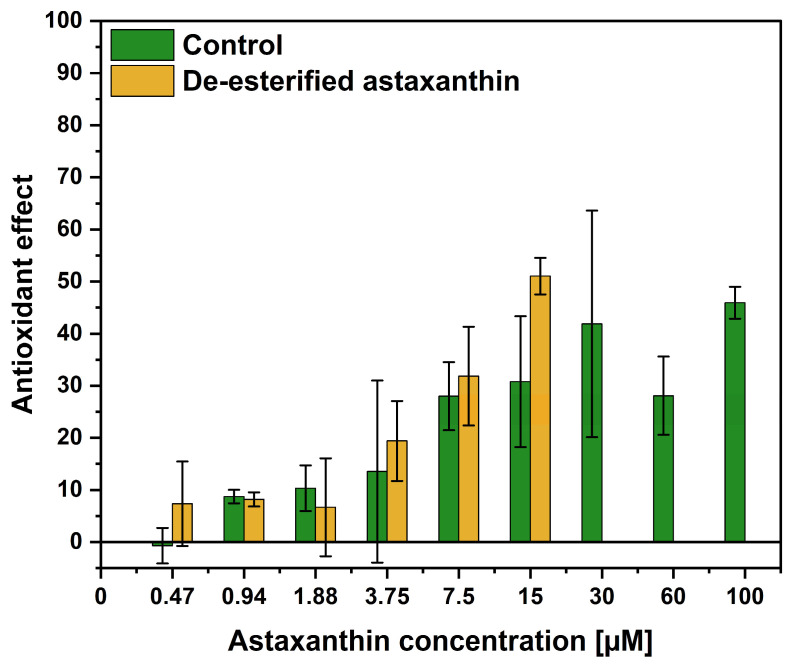
Intracellular antioxidant activity of de-esterified astaxanthin. Extract from *H. pluvialis* was treated with BSA (control; shown in green) or cholesterol esterase (de-esterified; shown in yellow). Values are given as mean ± standard deviation (n = 3).

We aimed to de-esterify the astaxanthin-(di)esters in the algal-based oleoresin in order to test the activity of an algal-derived free-form astaxanthin. Therefore, astaxanthin derived from *H. pluvialis* was de-esterified using cholesterol esterase, as confirmed by HPLC analysis (Figure S1). The AOP1 assay, which compared the de-esterified and the esterified astaxanthin, was performed as previously described, and the results are depicted in Figure 2. No statistical differences were observed between the control and de-esterified samples. Notably, the samples containing the de-esterified astaxanthin exhibited cytotoxic effects at concentrations > 15 µM, leading to incomplete results.

## 3. Discussion

This work demonstrates that the source and molecular form of astaxanthin significantly impact its antioxidant activities within cultured human keratinocytes. Astaxanthin-containing corynebacterial oleoresin exhibited a pronounced intracellular antioxidant effect, with an EC_50_ of 2.7 µM.

In a previous study, we have shown that corynebacterial and algal astaxanthin exhibit comparable antioxidant activities using the widely accepted DPPH assay [33]. While natural corynebacterial and algal astaxanthins showed a comparable EC_50_ of 3.2–3.7 µg mL^−1^, the racemic mixture of the synthetic astaxanthin had approximately 10-fold lower antioxidant activity (correspondingly a higher EC_50_ of 42 µg mL^−1^) [33]. These findings are consistent with previous reports indicating that the enantiomer pure (3S,3′S) astaxanthin exhibits a higher antioxidant activity than the (3R,3′R) enantiomer and the synthetic racemic mixture in different chemical assays such as ABTS and ORAC, as well as in cell- and organism-based in vivo studies [52].

The discrepancy between DPPH-based antioxidant activity and the intracellular AOP1 assay highlights the importance of complementing chemical assays for specific application evaluations [15]. While chemical antioxidant assays excel at quantifying radical-scavenging capacity in cell-free solutions, they lack information on bioavailability and cell uptake, which are practical hurdles that influence the antioxidant activity in cellular systems [14,53].

Astaxanthin itself is a lipophilic compound [44], but the solubility and bioavailability of the esterified (algal) and free form (corynebacterial and synthetic) astaxanthins [54], as well as the enantiomer composition, are considered to affect the stability and bioavailability [55,56]. In vitro and in vivo studies have shown that free astaxanthin is absorbed faster, since astaxanthin esters must be hydrolyzed by digestive enzymes and fluids prior to uptake [56,57,58].

As the conditions of the AOP1 assay do not favor ester hydrolysis, it is tempting to speculate that the algal-derived astaxanthin was not properly taken up by the HaCaT cells. A similar situation may apply to the cosmetic use of algal-derived astaxanthin: unlike free astaxanthin, the esterified form of algal astaxanthin is less bioavailable to the skin due to the absence of hydrolyzing enzymes, which makes it less suitable for topical applications [39,56].

Therefore, it was questioned whether the intracellular antioxidant activity of the algal-based astaxanthin could be increased after de-esterification. Although the de-esterification was successfully validated by HPLC analysis (Figure S1), the intracellular antioxidant activity in cultured keratinocytes did not differ from that of the control algal-based oleoresin. Cytotoxic effects of the de-esterified astaxanthin were observed at concentrations > 15 µM, leading to incomplete results. This cytotoxicity may be due to the release of free fatty acids during the de-esterification, which are known to exhibit in vitro cytotoxicity for cell cultures [59,60].

Astaxanthin exhibits skin-protective effects in a concentration-dependent manner. Cellular in vitro studies typically report efficacy in the low micromolar range (1–10 µM), while topical formulations commonly contain around 0.1%, and oral supplementation is usually applied at doses of 4–12 mg/day [61,62]. Overall, astaxanthin is considered a safe ingredient for both topical and oral use. At the concentrations typically used for topical application, adverse effects such as skin discoloration are unlikely to occur. Safety assessments by the European Food Safety Authority, as well as human intervention studies involving daily doses of 8–12 mg, have not identified any toxicological concerns [62,63,64]. Nevertheless, high concentrations of carotenoids may influence gastrointestinal function and lipid metabolism and can facilitate pro-oxidative effects under certain conditions [65]. Currently, there is no evidence of isomer-specific adverse dermatological or systemic effects of astaxanthin in humans. This indicates that differences between astaxanthin enantiomers primarily affect bioavailability and biological activity rather than safety. While synthetic astaxanthin is registered and widely used as a feed colorant in aquaculture, only natural astaxanthin derived from algae has been registered as a novel food for human consumption.

Despite the clear activity differences observed, some limitations should be considered. As the corynebacterial astaxanthin was tested as part of the oleoresin rather than as a purified compound, the synergistic effects of co-extracted compounds cannot be excluded. The oleoresin matrix itself may account for the superior activity, as byproducts in the extract may promote the solubility and/or delivery of the lipophilic astaxanthin [66]. It is known that lipid-rich matrices and formulation strategies can improve the bioavailability of carotenoids as compared to purified compounds [50,57,67]. Additionally, microneedle-based delivery systems can bypass the outermost layer of the skin, thereby potentially facilitating transdermal absorption [68].

In this study, the corynebacterial oleoresin was demonstrated to be the most promising source of astaxanthin for the intracellular ROS-scavenging in keratinocytes. The cell-based assay was developed by Gironde et al. (2020), who obtained the similar partial activity of synthetic astaxanthin [51] as observed in this study. Although the synthetic astaxanthin was most likely taken up by the cells due to its free form, the low activity was likely due to its less active enantiomers. When compared to the results of Gironde et al. 2020 [51], it should be noted that the astaxanthin-rich corynebacterial oleoresin is one of the most effective antioxidants identified in the AOP1 assay, with an EC_50_ of 2.7 µM in HaCaT cells (Figure 1).

As these results highlight the significance of alternative antioxidant resources from microbial fermentation [26], the real-world applicability should be further evaluated in other cell lines and skin penetration assays to strengthen the conclusion [50]. From an application perspective, *C. glutamicum* is a platform system offering access to other astaxanthin derivatives, such as astaxanthin-diglucoside [32], which may exhibit distinct bioactivities compared to the here tested astaxanthin variants.

## 4. Materials and Methods

### 4.1. Production and Extraction of Corynebacterial Astaxanthin

Astaxanthin-producing *C. glutamicum* strain ASTA** was cultivated for 48 h in shake flasks as described by [32] using the optimized trace element solution [31]. After cultivation, the biomass was harvested by centrifugation at 10,000× *g* for 20 min. Subsequently, the cell pellet was extracted using 90% (*v v*^−1^) ethanol at a solvent-to-biomass ratio of 15 mg_CDW_ mL^−1^ at 60 °C for 30 min in a 1 L stirred bottle reactor equipped with an anchor stirrer (DWK Life Sciences, Mainz, Germany) at 500 rpm. The astaxanthin oleoresin was prepared as described in [33] and was stored at −20 °C until further usage. The astaxanthin content in the oleoresin was 11.6 mg g^−1^ as determined by HPLC [32].

### 4.2. Intracellular In Vivo Antioxidant Assay

For the intracellular in vivo antioxidant assay (AOP1), the corynebacterial astaxanthin (as oleoresin) (CA) was compared to two other astaxanthin sources: synthetic astaxanthin (SA) (Sigma-Aldrich, St. Louis, MA, USA; catalog number 1044200) and esterified astaxanthin (as oleoresin; containing 100 mg g^−1^ astaxanthin (calculated as free astaxanthin); astaxanthin is present as 75% monoester, 20% diester, and 5% free) from *Haematococcus pluvialis* (AA) (Sigma-Aldrich, St. Louis, MA, USA; catalog number 1044210). All samples were dissolved in DMSO to a concentration of 3 mM (calculated as free astaxanthin) at 40 °C and 1000 rpm (Thermomixer comfort, Eppendorf, Wesseling, Germany). Stock solutions were shipped frozen to Antioxidant Power (Toulouse, France). Nine different astaxanthin concentrations were obtained by serial factor 2 dilutions, with the highest concentration of astaxanthin in the cell culture medium being 120 µM, corresponding to 4% DMSO. The assay was performed as described by [51] using human keratinocytes (HaCaT).

### 4.3. AOP1 Assay with De-Esterified Algal Astaxanthin

In order to verify that the low activity of the algal astaxanthin is based on the esters, a second AOP1 assay was performed, comparing both the free and the esterified astaxanthin from algae. To obtain the free version of the esterified astaxanthin from *H. pluvialis*, an enzymatic cleavage was performed, which was adapted from [69]. Per mL of acetone, 1.5 mg of *H. pluvialis* oleoresin was dissolved. The enzyme solution consisted of 2 U mL^−1^ cholesterol esterase from *Pseudomonas fluorescencs* (Sigma-Aldrich, St. Louis, MA, USA; catalog number: C9281) in 0.05 M Tris HCl, pH 7. The astaxanthin-containing acetone and the enzyme solution were mixed at a ratio of 3:2, and the reaction mixture was shaken at 37 °C in the dark for 4 h. The cholesterol esterase was replaced by bovine serum albumin (BSA) as a negative control. After incubation, 5 mL of hexane was added to the reaction mixture and mixed vigorously. The hexane layer was transferred into a new tube and subsequently evaporated (Concentrator Plus, Eppendorf, Germany). Successful de-esterification (>99%) was verified by HPLC (Figure S1). BSA-treated samples (control) and the cholesterol esterase-treated samples (de-esterified astaxanthin) were dissolved in DMSO at concentrations of 2.5 mM and 2.9 mM (calculated as free astaxanthin), respectively.

### 4.4. Quantification of Astaxanthin by HPLC

Astaxanthin was quantified by using the Agilent 1200 series (Agilent Technologies, Santa Clara, CA, USA) equipped with a reversed-phase precolumn (LiChrospher 100 RP18 EC-5, 40 × 4 mm) (CS-Chromatographie, Langerwehe, Germany), a reversed-phase main column (LiChrospher 100 RP18 EC-5, 125 × 4 mm) (CS-Chromatographie, Langerwehe, Germany), and a diode array detector (DAD) recording the absorption at λ = 470 nm. A defined amount of sample was dissolved in a 7:3 methanol:acetone mixture, of which 50 µL was injected. The mobile phases consisted of methanol:water (9:1) (A) and methanol (B). A gradient at a flow rate of 1.5 mL min^−1^ was used as follows: 0 min B: 0%, 10 min B: 100%, and 32.5 min B: 100%. Synthetic astaxanthin was used as a reference standard.

## 5. Conclusions

In conclusion, the results demonstrate that the astaxanthin-rich corynebacterial extract exhibits superior intracellular antioxidant activity in keratinocytes compared to algal and synthetic forms, highlighting the importance of molecular form and stereoisomer composition for cellular efficacy. Effective doses observed here for antioxidation in keratinocytes (EC_50_ of 2.7 µM) are consistent with concentrations reported in the literature. It can be hypothesized that the differences between tested astaxanthin variants primarily affect bioavailability and efficacy rather than safety.

## Data Availability

The original contributions presented in this study are included in the article/Supplementary Materials. Further inquiries can be directed to the corresponding author.

## Supplementary Materials

The following supporting information can be downloaded at: https://www.mdpi.com/article/10.3390/ijms27083638/s1.

## Abbreviations

The following abbreviations are used in this manuscript:

AOP	Antioxidant power test
AA	Algal astaxanthin
CA	Corynebacterial astaxanthin
CDW	Cell dry weight
SA	Synthetic astaxanthin
