# Supplementary material for: Superior Intracellular Antioxidant Activity of an Astaxanthin-Containing Corynebacterial Extract
Source: Int J Mol Sci. 2026 Apr 19;27(8):3638. doi: 10.3390/ijms27083638 (PMC13115732; doi:10.3390/ijms27083638)
Supplement: Supplementary file 1 [file ijms-27-03638-s001.zip › ijms-4260047-supplementary.pdf]

Article

Superior Intracellular Antioxidant Activity of an Astaxanthin-Containing Corynebacterial Extract

Jan Seeger [1] and Nadja A. Henke [2,*]

[1] Genetics of Prokaryotes, CeBiTec & Faculty of Biology, Bielefeld University, 33615 Bielefeld, Germany
[2] Institute of Process Engineering in Life Sciences, Karlsruhe Institute of Technology, 76131 Karlsruhe, Germany
* Correspondence: nadja.henke@kit.edu

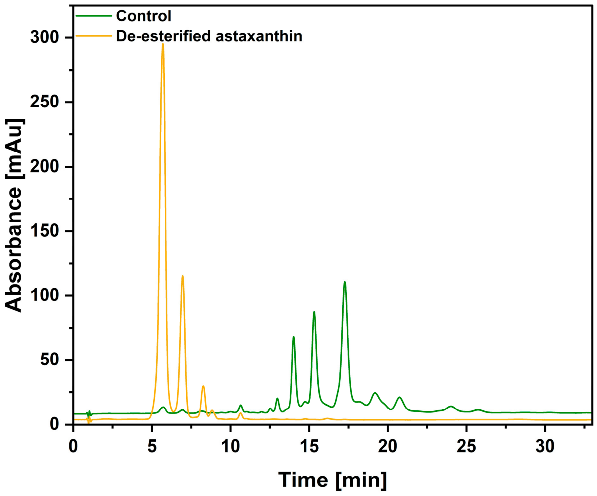

Figure S1. HPLC chromatogram of de-esterified astaxanthin. Extract from *H. pluvialis* was treated with BSA (control) or cholesterol esterase (de-esterified).

Academic Editor: Michele Mari

Received: 30 March 2026
Revised: 10 April 2026
Accepted: 15 April 2026
Published: date

Copyright: © 2026 by the authors. Submitted for possible open access publication under the terms and conditions of the Creative Commons Attribution (CC BY) license.
